# Use of non-HIV medication among people living with HIV and receiving antiretroviral treatment in Côte d’Ivoire, West Africa: A cross-sectional study

**DOI:** 10.1371/journal.pone.0221335

**Published:** 2019-09-16

**Authors:** Mariam Mama Djima, Didier Koumavi Ekouevi, Jean-Pierre Gregoire, Boris Tchounga, Patrick Ahuatchi Coffie, Viet-Thi Tran, Franck Y. Touré, Jocelyne Moisan

**Affiliations:** 1 PACCI, CHU Treichville, Abidjan, Côte d’Ivoire; 2 Institut Pasteur de Côte d’Ivoire, Abidjan, Côte d’Ivoire; 3 Faculté de pharmacie, Université Laval, Québec, Canada; 4 Centre Inserm U 1219, ISPED, Université Victor Segalen, Bordeaux, France; 5 Université de Lomé, Département de Santé Publique, Lomé, Togo; 6 Axe Santé des populations et pratiques optimales en santé, Centre de recherche du CHU de Québec–Université Laval, Québec, Canada; NPMS-HHC CIC / LSH&TM, UNITED KINGDOM

## Abstract

**Background:**

In Côte d’Ivoire, people living with HIV (PLHIV) have free access to antiretroviral therapy (ART) and cotrimoxazole. Yet, they may use other medications to treat non-HIV diseases. Scarce data are available regarding the use of non-HIV medications in Africa. This study describes the use of non-HIV medications and identifies the factors associated with their use by PLHIV on ART in Côte d’Ivoire.

**Methods:**

A cross-sectional study was conducted in six HIV clinics in 2016. HIV-1-infected adults receiving ART for at least one year were eligible. A standardized questionnaire was used to collect demographics, HIV characteristics and medication use data. Associated factors were identified using a multivariate adjusted Poisson regression.

**Results:**

A total of 1,458 participants (74% women) were enrolled. The median age was 44 years, and the median duration of ART was 81 months. A total of 696 (48%) participants reported having used at least one non-HIV medication. Among the 1,519 non-HIV medications used, 550 (36%) had not been prescribed and 397 (26%) were from the nervous system class. Individuals who were more likely to report the use of at least one non-HIV medication included those who had been treated in an Abidjan HIV clinic, had a high school education level, had a monthly income between 152 and 304 euros, had a poor perceived health status, had WHO advanced clinical stage, had used traditional medicine products and had not used cotrimoxazole.

**Conclusion:**

Almost half PLHIV on ART reported using non-HIV medication. Further research is needed to assess whether the use of non-HIV medication is appropriate given about a third of those medications are not being prescribed.

## Introduction

The successful scale-up of antiretroviral therapy (ART) during the last two decades has changed the face of the HIV epidemic, with HIV infection now being considered a chronic disease [1]. Adequate treatment has led to reductions in AIDS-related mortality and morbidity [2, 3] and has also improved the life expectancy of people living with HIV (PLHIV), which is now almost identical to the life expectancy observed in the general population [4–6]. Nevertheless, increased life expectancy and aging come with new challenges in HIV management. In addition to AIDS-related diseases, PLHIV are exposed to non-HIV diseases that occur independently of the weakness of the immune system [7, 8]. Non-HIV diseases are mostly chronic diseases [7, 9–12]; yet, PLHIV in Africa also suffer from acute diseases, such as malaria and respiratory infections [13, 14].

In resource-limited countries, the Word Health Organization (WHO) recommends the initiation of an ART with a cotrimoxazole prophylaxis for every PLHIV [15]. In Côte d'Ivoire, ART is accessible nation-wide [16] and is free of charge in public pharmacies [17–19]. This is not the case for the therapeutic management of non-communicable diseases, such as diabetes, hypertension and cancer.

In low-income countries, non-HIV medications may be purchased with or without a prescription. Taking non-HIV medications without a prescription depends on many factors, such as previous successful experiences with the same or similar medications, the cost of non-HIV disease management [19, 20], non-HIV essential medications not usually being available in public health care settings [20], the accessibility of health facilities [21–24], insufficient health insurance coverage [25, 26] and perceiving that the disease is not serious [21–24]. When the use of non-prescribed medications is unjustified and inappropriate, there is an increased risk of drug-drug interactions [27, 28], medication resistances, undesirable side effects and increased health expenditures [29, 30].

There is limited information on the use of non-HIV medications among PLHIV. To our knowledge, seven studies have focused on the use of non-HIV medications among PLHIV in Africa [19, 26, 31–34]. In three of those studies, non-HIV medications were only listed [26, 32, 33], and in another one, the focus was on the cost of non ARV medications [19]. Only two studies, one from Senegal [31] and the other from South Africa [34], reported the proportions of PLHIV on ART who used at least one non-HIV medication (85% and 87%, respectively). In these studies, the patients included were on ART less than one year and data was not available on non HIV medication. Moreover, no distinction was made between non-HIV prescribed medications and non-HIV non-prescribed medications. To our knowledge, the use of non-HIV medications either prescribed or not has not been comprehensively studied in Africa. Our study aimed to describe the use of non-HIV medications among PLHIV on ART for at least 12 months in Côte d’Ivoire and to identify factors associated with the use of at least one non-HIV medication.

## Materials and methods

### Study design and settings

A cross-sectional study was conducted from April 11th to September 28th, 2016, in six HIV clinics of Côte d’Ivoire. Three of these clinics were located in Abidjan, the economic capital, one in San Pedro in the South-west of the country, one in Bouaké in the Center of the country and the last in Korhogo in the North. These cities were selected in regions with high HIV prevalence. In Abidjan, the three largest HIV clinics were selected. In the three other regions, we selected the clinic with the largest number of PLHIV.

### Study population and enrollment of patients

The study population included individuals living with HIV-1, aged 18 years or older, receiving ART for at least one year. Every day during the study period, all eligible individuals attending the participating HIV clinics for a routine follow-up visit were offered to participate in the study. After confirming their eligibility in the medical patient record, the site investigator provided clear information on the study, discussed the information sheet with the participant and fixed an appointment for those who agreed to participate. Three days before the appointment date, each participant received a reminder call from a social worker to confirm the time of the interview.

### Data collection

On the day of interview, the participant signed the consent form before the interview began. During the interview, a social worker collected each participant’s demographics and HIV characteristics (WHO clinical stage, history of CD4 count, clinical events, ART regimen and other treatments) using a standardized questionnaire formatted as a case report file (CRF). Data on all medications and traditional and complementary medicine (TCM) products used during the preceding 30 days were also collected. To help in identifying medications used, participants were asked to bring all medications they used. For participants who did not bring their medications, a photo library was shown to help them identify medications they were using. A pharmacist analyzed pictures of all the material brought by the participants (containers, prescription forms, and products) to determine the names, formulations and doses of all medications used by the participants.

### Outcome variable

The main outcome was the use of at least one non-HIV medication. Participants were considered to use at least one non-HIV medication if they reported using any medication other than antiretroviral medications and cotrimoxazole.

### Independent variables

Patient-related variables included demographic characteristics such as age, sex, have a significant other, education level, being employed or not, monthly income in euros and health insurance coverage. In the context of HIV infection, the term “older” refers to patients aged ≥ 50 years [35, 36]. A participant was considered to have health insurance coverage if he/she reported having medical or/and pharmaceutical insurance coverage. Region was defined according to the localization of the recruiting HIV center: economic capital (for clinics in Abidjan) or hinterland (for clinics in Bouaké, San Pedro and Korhogo).

Health-related variables consisted of data on perceived health status, alcohol consumption and HIV characteristics. Perceived health status was measured using a 6-level Likert scale (excellent to poor). Perceived health status was considered “at least acceptable” when the response was either excellent, very good, good or acceptable; otherwise, it was considered “poor”. Alcohol consumption was measured using questions from the WHO STEPS questionnaire (37). Two categories were defined. Alcohol users were those who reported they had used alcohol in the 30 days prior to the interview. The following HIV characteristics were abstracted from the medical records: the date of HIV diagnosis, the initial CD4 count in cells/mm^3^ (< 200, 200–349, 350–499, ≥ 500), and the WHO clinical stage at diagnosis. The duration of HIV infection was the number of months between the date of HIV diagnosis and the date of the interview.

Concerning treatment-related variables, we measured the use of HIV and non-HIV medications along with traditional and complementary medicines. For HIV medications, current use of ART and cotrimoxazole was abstracted from medical records. ART was classified into two categories: first-line ART (either three nucleoside reverse transcriptase inhibitors or two nucleoside reverse transcriptase inhibitors with a non-nucleoside reverse transcriptase inhibitor) and second- or third-line ART (two nucleoside reverse transcriptase inhibitors with a protease inhibitor or with an integrase inhibitor) [37, 38]. The duration of ART in months was defined as the time between the date of ART initiation and the date of the interview. Each participant was asked to report the number of ART pills taken each day. Finally, participants were considered to have used a TCM product if they reported having used a TCM within the last 30 days. TCM products were from two main TCM therapeutic modalities: 1) TCM consisting of spiritual and manual treatments, 2) TCM consisting of herbal medicines, veterinary medicines and / or mineral medicines [39].

Non-HIV medications were classified according to the WHO Anatomical Therapeutic Chemical Classification (ATC) system [40]. For each non-HIV medication, the pharmacological classes were determined. Medications that could not be identified due to a lack of information (such as medications bought in a public market and medications with information written only in Arabic or Chinese) were coded 99999. Natural products that did not have a code in the ATC classification were coded 88888. When a drug had more than one ATC code, we used the code relevant to the self-reported illness. For the choice of ATC codes for generic medications whose International Common Denominations (ICDs) were difficult to identify, we used the national list of the reimbursable medications from ministry of health that contains generic medication names associated with the ICDs for medications.

Participants were considered to have used a TCM product if they reported having used a TCM within the last 30 days.

### Statistical analysis

Statistical analyses were performed using SAS version 9.4 (SAS Institute, Cary, NC). Pearson Chi square tests were used to compare proportions, Student’s t-tests were used to compare means and Wilcoxon tests were used to compare medians. Factors associated with the use of at least one non-HIV medication were identified using a multivariate Poisson regression model. We calculated prevalence ratios (PRs) with their 95% confidence intervals (CIs) (32). To build the model, we included all variables associated with the use of at least one non-HIV medication in the unadjusted analysis (*P* < 0.2) and used the backward method to identify only variables independently predictive of the use of at least one non-HIV medication with a *P*-value < 0.05. Sex and age were forced into the final model (33, 34).

### Ethical consideration

Before being enrolled in the study, each participant received clear information and provided written consent to participate, including the authorization to use personal data for research purposes. The study was approved by the National Ethics Committee for Research in Côte d’Ivoire and the ethics in research committee of CHU de Quebec-Université Laval.

## Results

A total of 1,729 HIV patients were approached to participate in the study. Among them, 1,458 (84%) agreed and were included. The reasons for non-participation are summarized in Table 1.

**Table 1:** Frequency of reported reasons to decline participation (N = 271).

Reasons	N	%
Were unavailable for the interview	88	32.5
Could not be reached	41	15.1
Gave no reason to decline participation	20	7.4
Were not interested by the study	20	7.4
Were outside the city during the study period	19	7.0
Did not come to the interview	19	7.0
Either had or a family member had already participated in another study	14	5.2
Did not use modern and traditional medicine	13	4.8
Were not called to make an appointment	9	3.3
Had other reasons*	28	10.3
Total	**271**	100

* accident, need approbation from family, fear of breaking confidentiality, financial difficulties

Characteristics of the participants according to the region where they were recruited are presented in Table 2. They had a median age of 44 years, IQR (38–50), 27% were aged ≥ 50 years and 736 (50%) were recruited in Abidjan. Approximately 75% of the participants were women. Among the participants, 145 (10%) had health insurance coverage, of which 82% lived in Abidjan. In addition, 776 (53%) reported having used at least one TCM product in the 30 days preceding the interview. Participants attending an HIV clinic in Abidjan had a higher level of education and a higher income than the participants attending a hinterland HIV center.

**Table 2:** Characteristics of the 1458 participants according to the recruitment setting.

Characteristics	Total N = 1458		Abidjan HIV clinics N = 736		Hinterland HIV clinics N = 722		Pearson chi-squared (χ2) test^$^/ Wilcoxon test^£^
	N	%	n	%	n	%	*P* values
**Patient-related** Age (years)							
Median [IQR]	44	[38–50]	44	[39–51]	44	[37–50]	0.048
< 50 years	1070	73.4	525	71.9	541	74.9	00.18
≥ 50 years	388	26.6	207	28.1	181	25.1	
Sex							
Men	381	26.1	198	26.9	183	25.3	0.499
Women	1077	73.9	538	73.1	539	74.7	
Body Mass index							
Underweight (< 18.5)	165	11.3	71	09.6	94	13.0	<0.0001
Normal weight (18.5–24.99)	796	54.6	373	50.7	423	58.6	
Overweight (25–29.99)	359	24.6	205	27.9	154	21.3	
Obese (≥ 30)	138	09.5	87	11.8	51	07.1	
Have a significant other							
Yes	726	49.8	360	48.9	366	50.7	0.496
No	732	50.2	376	51.1	356	49.3	
Education							
No school	454	31.1	142	19.3	312	43.4	<0.0001
Primary level	433	29.7	217	29.5	216	29.7	
High school	470	32.2	290	39.4	180	24.9	
> High school	101	6.93	87	11.8	14	01.9	
Employment							
Yes	1179	80.9	620	84.2	559	77.4	0.0009
No	279	19.1	116	15.8	163	22.6	
Monthly income in euros							<0.0001
< 92	964	66.1	364	49.4	600	83.1	
92–151	235	16.1	183	24.9	52	07.2	
152–304	148	10.2	105	14.3	43	06.0	
≥ 304	90	06.2	79	10.7	11	01.5	
Don’t Know	21	01.4	05	00.7	16	02.2	
Health insurance coverage							
Yes	145	10.0	119	16.2	26	03.6	<0.0001
No	1313	90.0	617	83.8	163	96.4	
**Health related**							
Perceived Health Status							
At least acceptable	1166	80.0	610	82.9	556	77.0	0.0038
Poor	281	19.3	120	16.3	161	22.3	
Missing data	11	00.7	06	00.8	05	00.7	
Hospitalization in the year before interview							
Yes	116	09.9	63	08.6	53	07.3	00.39
No	1313	90.1	673	91.4	696	92.7	
Alcohol consumption							
Yes	361	24.8	284	38.6	77	10.7	<0.0001
No	1096	75.2	451	61.4	645	89.3	
HIV duration (months); median [IQR]	91	[57–120]	95	[58–129]	87	[55–113]	<0.0001
≤ 120	1096	75.2	511	69.4	585	81	<0.0001
>120	362	24.8	225	30.6	137	19	
Antiretroviral treatment (ART) duration (months); median [IQR]	81	[47–110]	82	[47–116]	80	[44–113]	<0.0001
≤ 120	1206	82.7	570	77.4	636	88.1	<0.0001
>120	252	17.3	166	22.6	86	11.9	
Initial CD4 count in cells/mm3; median [IQR]							
< 200	685	47.0	319	43.3	366	50.7	0.0001
200–349	393	27.0	198	26.9	195	27.0	
350–499	218	14.9	138	18.8	80	11.1	
≥ 500	138	09.5	78	10.6	60	08.3	
Missing data	24	01.6	03	00.4	21	02.9	
	N	%	n	%	n	%	*P* values
WHO clinical stage							
I	535	36.7	255	34.6	280	38.8	<0.0001
II	521	35.7	187	25.4	334	46.3	
III or IV	380	26.1	276	37.5	104	14.4	
Missing data	01	0.1	00	00	01	0.1	
Data not available	21	1.4	18	2.4	03	0.4	
**Treatment-related**							
ART regimen							<0.0001
3 NRTIs^§^ / NNRTIs^μ^+2 NRTIs	1182	81.1	538	73.1	644	89.2	
Others	276	18.9	198	26.9	78	10.8	
Daily ART pills median [IQR]	02	[1–10]	02	[1–5]	02	[2–3]	<0.0001
1	284	19.5	206	28.0	78	10.8	<0.0001
2	650	44.6	203	27.6	447	61.9	
[3–4]	261	17.9	139	18.9	122	16.9	
≥ 5	263	18.0	188	25.5	75	10.4	
Use of cotrimoxazole							
Yes	535	36.7	353	48.0	182	25.2	<0.0001
No	923	63.3	383	52.0	540	74.8	
Use of traditional and complementary products							
Yes	776	53.2	365	49.6	411	56.9	0.005
No	682	46.8	371	50.4	311	43.1	
Use at least one non-HIV medication							<0.0001
Yes	696	47.7	400	54.3	296	41.0	
No	762	52.3	336	45.7	426	59.0	

^£^Pearson Chi square tests were used to compare proportions

^$^Wilcoxon tests were used to compare medians

^§^NRTIs nucleoside and nucleotide reverse transcriptase inhibitors (NRTIs)

^μ^ NNRTIs Non-nucleoside Reverse Transcriptase Inhibitor, IQR: interquartile Range.

### HIV medications use

The median ART duration was 81 months (IQR: 47–110), with most participants (n = 1,182; 81%) on a first-line ART regimen. The median number of ART pills taken daily was 2 (IQR: 2–3), with 263 (18%) participants taking ≥ 5 ART pills. A total of 535 (37%) patients reported using cotrimoxazole in addition to ART, with the proportion of PLHIV using cotrimoxazole being higher in Abidjan than in the hinterland (48% vs. 25%, *P* = <0.0001).

### Non-HIV medications use

A total of 696 (48%) participants reported using at least one non-HIV medication. Among them, 319 (46%) reported using one medication, 169 (24%) two medications, 95 (14%) three medications and 113 (16%) ≥ four medications. The median number of non-HIV medications per person was two (IQR: 1–3). Among users of non-HIV medications, 284 (41%) used only non-prescribed medications (median: 1; IQR: 1–2), 318 (46%) used only prescribed medications (median: 2; IQR: 1–3) and 94 (13%) used both prescribed and non-prescribed medications (median: 3; IQR: 2–5). Concerning non-communicable disease, the proportions of users of non-HIV medications for the treatment of cardiovascular disease and diabetes were 5% (N = 68), and 1.1% (N = 16), respectively.

### Pharmacologic classes of non-HIV medications

Overall, the number of non-HIV medications reported to be used by the 696 participants totaled 1,519 (Table 3), of which 969 (64%) were prescribed. Among the 1,519 reported non-HIV medications, 397 (26%) were from the nervous system class (reported by 349 (24%) participants), and 260 (17%) were from the alimentary tract and metabolism class (reported by 214 (15%) participants). Among the 550 non-HIV non-prescribed medications, 210 (38%) were analgesics, with the majority being non-opioid medications (used by 177 (12%) participants), along with 47 (9%) antibacterial medications (used by 43 (3%) participants) and 44 (8%) anti-inflammatory and anti-rheumatic medications (used by 41 (3%) participants).

**Table 3:** Anatomical, Therapeutic and Chemical (ATC) classification of non-HIV medications used according to whether or not they were prescribed.

	Total (N = 1519)		Medication prescribed (n = 969)		Medication not prescribed (n = 550)	
	N	%	n	%	n	%
**A: Alimentary tract and metabolism**	**260**	**17.12**	**162**	**18.18**	**78**	**14.18**
A01: Stomatological preparations	07	0.46	07	0.31	00	0.00
A02: Medications for acid related disorders	33	2.17	24	2.48	09	1.64
A03: Drugs for functional gastrointestinal disorders	13	0.86	11	1.14	02	0.36
A04: Antiemetic and antinauseants	02	0.13	02	0.21	00	0.00
A05: Bile and live therapy	04	0.26	04	0.41	00	0.00
A06: Drugs for constipation	03	0.20	02	0.21	01	0.18
A07: Antidiarrheal, intestinal anti-inflammatory/anti-infective agents	09	0.59	07	0.72	02	0.36
A10: Medications used in diabetes	20	1.32	20	2.06	00	0.00
A11: Vitamins	60	3.95	41	4.2	19	3.45
A12: Mineral supplements	88	8.79	49	5.06	39	7.09
**B: Blood and blood forming organs**	**61**	**4.02**	**53**	**5.47**	**08**	**1.45**
B01: Antithrombotic agents	04	0.26	03	0.31	01	0.18
B03: Anti-anemic drugs	57	3.75	50	5.16	07	1.27
**C: Cardiovascular system**	**79**	**5.20**	**72**	**7.93**	**07**	**1.27**
C01: Cardiac therapy	07	0.46	06	0.62	10	0.18
C02: Antihypertensive	04	0.26	02	0.21	02	0.36
C03 Diuretics	05	0.33	05	0.52	00	00
C05: Vasoprotectives	07	0.46	05	0.52	02	0.36
C07: Beta blocking agents	12	0.79	12	1.24	00	0.00
C08: Calcium channel blockers	10	0.66	10	1.03	00	0.00
C09: Agents acting on the renin (angiotensin system)	24	1.58	24	2.48	00	0.00
C10: Lipid modifying agents	10	0.66	08	0.83	02	0.36
**D: Dermatologic drugs**	**24**	**1.58**	**18**	**1.86**	**6**	**1.09**
D01: Antifungals for dermatological use	06	0.39	05	0.52	01	0.18
D02: Emollients and protectives	01	0.07	01	0.10	00	0.00
D06: Antibiotics and chemotherapeutics for dermatological use	02	0.13	01	0.10	01	0.18
D07: Topical dermatological corticosteroids	09	0.59	06	0.62	03	0.55
D08: Antiseptics and disinfectants drugs	03	0.20	03	0.31	00	0.00
D10: Anti-acne preparations	01	0.07	0	0.00	01	0.18
D11: Other dermatological preparations	02	0.13	2	0.21	00	0.00
**G: Genitourinary system and reproductive hormones**	**24**	**1.58**	**19**	**1.96**	**05**	**0.91**
G01: Gynecological anti-infectives and antiseptics	09	0.59	07	0.72	02	0.36
G03: Sex hormones and modulators of the genital system	14	0.92	11	1.14	03	0.55
G04: Urologicals	01	0.07	01	0.10	00	0.00
**H: Systemic hormonal preparations, excluding reproductive hormones and insulins**	**10**	**0.66**	**09**	**0.93**	**01**	**0.18**
H02: Corticosteroids systemic	10	0.66	09	0.93	01	0.18
**J: Anti-infectives for systemic use**	**144**	**9.48**	**97**	**10.01**	**47**	**8.55**
J01: Antibacterial drugs	135	8.89	88	9.08	47	8.55
J02: Antimycotic drugs	07	0.46	07	0.72	00	0.00
J05: Antivirals for systemic use	02	0.13	02	0.21	00	0.00
**M: Musculoskeletal system**	**114**	**7.50**	**68**	**7.02**	**48**	**8.36**
M01: Anti-inflammatory and antirheumatic drugs	100	6.58	56	5.78	44	8.00
M02 Topical products for joint and muscular pain	04	0.26	03	0.31	01	0.18
M03: Muscle relaxants	08	0.53	07	0.72	01	0.18
M09: Other drugs for disorders of the musculo-skeletal system	02	0.13	02	0.21	00	0.00
**N: Nervous system**	**397**	**26.14**	**183**	**18.09**	**214**	**38.91**
N02: Analgesic drugs	367	24.16	157	16.20	210	38.18
N03: Antiepileptics	03	0.20	03	0.31	00	0.00
N05: Psycholeptics drugs	14	0.92	11	1.14	03	0.55
N06: Psychoanaleptics	11	0.72	10	1.03	01	0.18
N07: Other nervous system drugs	02	0.13	02	0.21	00	0.00
**P: Antiparasitic products, insecticides and repellents**	**165**	**10.86**	**118**	**12.18**	**47**	**8.55**
P01: Antiprotozoal drugs	130	8.56	92	9.49	38	6.91
P02: Anthelmintic drugs	35	2.30	26	2.68	09	1.69
**R: Respiratory system**	**159**	**10.47**	**102**	**10.53**	**57**	**10.36**
R01: Nasal preparations	45	2.96	26	3.45	19	2.68
R02: Throat drugs	03	0.20	03	0.31	00	0.00
R03: Drugs for obstructive airway diseases	09	0.59	07	0.72	02	0.36
R05: Cough and cold drugs	35	2.30	25	2.58	10	1.82
R06: Antihistamines for systemic use	64	4.21	41	4.23	23	4.18
R07/ Other respiratory system products	03	0.20	00	0.00	03	0.55
**S: Sensory organs**	**30**	**1.97**	**27**	**2.79**	**03**	**0.55**
S01: Ophthalmological drugs	27	1.78	25	2.58	02	0.36
S02: Otologicals	03	0.20	02	0.21	01	0.18
**V: Various ATC structures**	**4**	**0.26**	**4**	**0.41**	**0**	**0.00**
V06: General nutrients	4	0.26	4	0.41	0	0.00
**Medication not identified**	**37**	**2.44**	**23**	**4.18**	**14**	**1.44**

### Factors associated with the use of at least one non-HIV medication

In the multivariate Poisson regression, after adjusting for age and sex, seven factors were statistically associated with the use of non-HIV medication (Table 4). Individuals who had a high school education level (PR = 1.17 [95% CI = 1.02–1.35] vs. unschooled), a monthly income between 152 and 304 euros (PR = 1.31 [95% CI = 1.12–1.53] vs. < 92euros), a poor (vs. at least acceptable) perceived health status (PR = 1.30 [95% CI = 1.06–1.46]), a WHO clinical stage II or III/IV (PR = 1.24 [95% CI = 1.08;1.41]; PR = 1.22 [95% CI = 1.06;1.40]), and those who were TCM product users (vs. no (PR = 1.22 [95% CI = 1.10–1.37]) were more likely to report the use of at least one non-HIV medication. Participants recruited in a hinterland HIV clinic (PR = 0.72 [95% CI = 0.64–0.82]) and cotrimoxazole users (vs. not) (PR = 0.77 [95% CI = 0.67–0.84]) were less likely to report the use at least one non-HIV medication.

**Table 4:** Associations between patient-related, health-related and treatment-related characteristics and use of at least one non-HIV medication (N = 1458).

Characteristics	Use of at least one non-HIV medication				Crude prevalence ratios	95% Confidence Intervals (CI)	*P* values	Adjusted prevalence ratios	95% CI	*P* values
	Yes		No							
**Patient-related**										
Age (years)										
< 50	518	74.4	552	72.4	0.96	[0.85–1.10]	0.40	0.90	[0.84–1.09]	0.59
≥ 50	178	25.6	210	27.6	1	1		1	1	
Sex										
Men	173	24.9	208	27.3	1.07	[0.94–1.21]	0.30	1.12	[0.97–1.29]	0.12
Women	523	75.1	554	72.7	1					
HIV clinics										
Hinterland	296	42.5	426	55.9	0.75	[0.68–0.84]	<0.001	0.72	[0.64–0.82]	<0.001
Abidjan	400	57.5	336	44.1	1					
Have a significant other										
Yes	362	52.0	364	47.8	1					
No	334	48.0	398	52.2	0.91	[0.82–1.01]	0.10			
Education										
No school	184	26.4	270	35.4	1					
Primary level	199	28.6	234	30.7	1.13	[0.97–1.32]	0.103	1.04	[0.89–1.21]	0.62
Secondary school	249	35.8	221	29.0	1.31	[1.14–1.50]	0.0002	1.17	[1.02–1.35]	0.026
> High school	94	9.2	37	4.9	1.56	[1.30–1.88]	<0.0001	1.23	[0.99–1.50]	0.055
Monthly Income in Euros										
< 92	437	62.8	527	69.2	1			1		
92–151	107	15.4	128	16.8	1.00	[0.86–1.17]	0.95	0.91	[0.77–1.06]	0.24
152–304	92	13.2	56	7.3	1.37	[1.19–1.58]	< 0.0001	1.31	[1.12–1.53]	0.0006
≥ 304	52	7.5	38	5.0	1.27	[1.05–1.54]	0.01	1.11	[0.88–1.41]	0.36
Don’t Know	8	1.1	13	1.7						
Insurance coverage										
Yes	88	12,6	57	7.5	1.31	[1.14–1.51]	0.0002			
No	608	87.4	705	92.5	1					
**Health-related**										
Perceived Health Status										
Poor	500	71.9	596	78.2	1.38	[1.23–1.55]	< 0.0001	1.30	[1.16–1.46]	<0.0001
At least acceptable	195	28.0	166	21.8	1					
Missing data	1	0.1								
Time since HIV diagnosis (months)	96	12–235	85	12–270						
≤ 120	498	71.5	598	78.5	0.83	[0.74–0.93]	0.0014			
> 120	198	28.5	164	21.5						
Initial CD4 count in cells/mm^3^;										
< 200	334	48.0	351	46.0	1.004	[0.83–1.21]	0.96			
200–349	186	26.7	207	27.2	0.97	[0.78–1.19]	0.80			
350–499	96	13.8	122	16.0	0.90	[0.72–1.14]	0.40			
≥ 500	67	9.6	71	9.3	1					
Missing data	13	1.9	11	1.5						
WHO HIV clinical stage										
I	219	31.5	316	41.5	1					
II	265	38.0	256	33.6	1.24	[1.09–1.42]	0.0013	1.24	[1.08–1.41]	0.0017
III or IV	205	29.5	205	23.0	1.32	[1.15–1.81]	<0.0001	1.22	[1.06–1.40]	0.005
Missing data	0	00.0	1	00.1						
Data not available	7	01.0	14	01.8						
**Treatment-related**										
Use of cotrimoxazole										
No	462	66.4	461	60.5	1					
Yes	234	33.6	301	39.5	0.87	[0.78–0.98]	0.022	0.77	[0.69–0.87]	<0.0001
Use of traditional and complementary products										
Yes	407	58.5	369	48.4	1.24	[1.11–1.38]	0.0001	1.22	[1.10–1.37]	<0.0001
No	289	41.5	393	51.6	1					

## Discussion

This is one of the first studies performed in West Africa describing the use of non-HIV medications among PLHIV on ART. In summary, 48% of PLHIV reported using at least one non-HIV medication, and 54% of them used non-prescribed drugs. The most reported classes of non-HIV medication used were the nervous system class (26%) and the alimentary tract and metabolism class (17%). Non-prescribed non-HIV medications represented 36% of 1,519 non-HIV medications, of which 39% were analgesic medications. Factors associated positively with the use of non-HIV medication were having a high level of school education, a monthly income between 152 and 304 euros, using TCM products, having a poor perceived health status and having a WHO stage II or III/IV. However, being treated in an HIV hinterland clinic and using cotrimoxazole were factors negatively associated with the use of non-HIV medication.

The proportion of PLHIV using non-HIV medications (48%) we observed was lower than those observed in most of the previous studies in both high- [41–50] and low-income countries [31, 34], where the proportions varied between 68% and 95%, distributed as follows: 68% in Switzerland [46, 50], 75% in Canada [45], 82% in Spain [42], 83% in USA [43], 85% in Senegal [31] 85% in USA [44], 87% in South Africa [34], 89% in Canada [41], 91% in Canada [47], 93% in USA [48] and 95% in Canada [49]. This difference might be explained by different reasons. Older PLHIV face many aging-related diseases [51, 52]. Our population was younger than those included in the high-income countries studies. Indeed, 32% [43, 47, 50] to 100% [42] of patients from high-income countries were over 50 years old, whereas in our study, only 27% were over 50 years old. Third, in the studies in high-income countries, 79% [43, 48] to 100% [45] of participants had public or private drug insurance, whereas only 10% of our study participants had such insurance, which may limit access to non-HIV medications.

One study conducted in Africa reported proportions of non-HIV medication users [31]. This was a retrospective study conducted in Senegal including 331 PLHIV who initiated ART between 2009 and 2011 and were followed until March 2012. PLHIV in the first year of ART initiation represented 43% of the participants. After a mean duration of 11.4 months on ART, 85% of patients received at least one prescription for a non-ART medication. In this study, the most frequently prescribed non-ART medications were cotrimoxazole (78.9% of patients), iron (33.2%), vitamins (21.1%) and antibiotics (19.6%).

Regarding non-HIV medication findings, in Côte d’Ivoire, before the availability of ART, in a cohort study, Nombela et al. reported 58,776 medications prescribed among 592 PLHIV. The most used classes of medication were the anti-infectious (32.8%) and pain medication (22.9%) classes [32]. In our study, the most common non-HIV medications used by participants were from the nervous system class (26%), as in some studies conducted in high-income countries [42, 47, 48]. However, we observed that the proportion of nervous system class medication users (24%) in our study was lower than the proportions observed in the USA (32%) [44], (52% [48]), Spain (44% [42]) and Canada (58% [47]). This difference could be explained by a greater diversity of therapeutic classes (antidepressants/antipsychotics/anxiolytics/analgesics) used in high-income countries, unlike in our study, where 96% of users of nervous system medications took analgesics. Indeed, accessibility to nervous system classes other than analgesics is limited in low-resource countries [53]. Furthermore, mental health services are only accessible in specialized health facilities.

The proportions of users of medications for the treatment of cardiovascular diseases and diabetes in our study were 5% and 1%, respectively, which are lower than those found in high-income countries (with the percentages of cardiovascular medication users being 43% [47], 26% [42], and 42% [44] and the percentage of diabetes medication users being 11% [44, 48]). This difference could be explained by the fact that diagnosis of non-communicable diseases in low-income countries is not routinely performed during HIV visits and by the low proportion of old PLHIV in our study. Therefore, we hypothesize that in our study, PLHIV with potentially non-communicable diseases may suffer from cardiovascular disease or diabetes, but they are not aware of their condition because they do not have access to diagnostics. The users of sex hormones and modulators of the genital system represented 9% [47] and 19% [44] in the PLHIV populations of high-income countries. In our study, only 0.9% used these drugs.

The use of a prescription medication without a prescription from a health professional was observed in our PLHIV population. Antibiotics and anti-malaria medications were used without prescription by 3.0% and 2.5% of participants, respectively. Individuals using those drugs without a prescription have likely not been exposed to appropriate diagnostic tests. The misuse of antibiotics or malaria medications can increase the risk of resistance to these medications and of drug-drug interactions [47, 54].

Seven factors were associated with the use of non-HIV medications. Some of these factors, such as WHO advanced clinical stage [31] and high level of education and high income [19], are in line with those that have been reported in previous studies. In our study, we observed that PLHIV living in Abidjan had higher incomes and access to non-HIV medications compared with PLHIV from the hinterland HIV clinics.

This study provides insight on the use of non-HIV medication by PLHIV on ART in West Africa. To our knowledge, this is the first study conducted in Africa that describes the extent to which non-HIV medications used were prescribed or not.

Our study has some limitations. First, we could not include patients who did not show up for their HIV follow-up. It is possible that these people were sicker than our participants. Therefore, we may have underestimated the proportion of non-HIV medication users. Second, the use of non-HIV medication was self-reported, which is susceptible to a memory bias. Therefore, the proportion of people using non-prescribed non-HIV medications may have been underestimated. Similarly, PLHIV who forgot their medications or prescriptions at home may have under-reported the use of non-HIV medications. If these medications were not actually medications, the proportion of non-HIV medications would be underestimated. Moreover, answers to questions related to income and health status perception may have suffered from social desirability bias. Next, since our study was conducted in only four regions in Côte d’Ivoire, our results are not generalizable to the entire population of PLHIV on ART in Côte d’Ivoire.

## Conclusion

In this study, we observed that approximately 50% of PLHIV on ART for at least one year used a non-HIV medication. More than half of them used non-HIV medications that were not prescribed. The results of this study suggest that healthcare professionals should pay attention to the needs of PLHIV in terms of non-HIV medications, particularly for patients living in Abidjan and those who are not using cotrimoxazole.

## Supporting information

S1 Database Database_MOTUHS. (DOCX)
