# Supplementary material for: Use of non-HIV medication among people living with HIV and receiving antiretroviral treatment in Côte d’Ivoire, West Africa: A cross-sectional study
Source: PLoS One. 2019 Sep 16;14(9):e0221335. doi: 10.1371/journal.pone.0221335 (PMC6746366; doi:10.1371/journal.pone.0221335)
Supplement: S1 Database — (DOCX) [file pone.0221335.s001.docx]

**Appendix 1. Sociodemographic characteristics of participants with missing data.**

**Appendix 1.** Sociodemographic and behavioral characteristics of participants who were included in the concurrency analysis (n=3,019), and those who were excluded from the analysis due to missing relational timing data (n=930). P-values compare the proportion between groups and were calculated with Chi-squared tests.


|  | Non-missing | | Missing | | p-value |
| --- | --- | --- | --- | --- | --- |
|  | N | (%) | N | (%) |  |
| Total | 3019 | (100) | 930 | (100) |  |
| HIV Status |  |  |  |  |  |
| Positive | 241 | (8.0) | 97 | (10.4) | 0.020 |
| Negative | 2778 | (92.0) | 833 | (89.6) |  |
| Previous HIV test |  |  |  |  |  |
| Yes | 1864 | (61.7) | 559 | (60.1) | 0.371 |
| No | 1155 | (38.3) | 371 | (39.9) |  |
| Location |  |  |  |  |  |
| Lima | 1477 | (48.9) | 646 | (69.5) | <0.001 |
| Outside Lima | 1542 | (51.1) | 284 | (30.5) |  |
| Age (years) |  |  |  |  |  |
| ≤21 | 809 | (26.8) | 275 | (29.6) | 0.331 |
| 22-25 | 632 | (20.9) | 194 | (20.9) |  |
| 26-31 | 664 | (22.0) | 202 | (21.7) |  |
| ≥32 | 914 | (30.0) | 259 | (27.8) |  |
| Any Post-Secondary Education | |  |  |  |  |
| Yes | 1168 | (61.3) | 290 | (31.2) | <0.001 |
| No | 1851 | (38.7) | 640 | (68.8) |  |
| Income |  |  |  |  |  |
| < Minimum Wage | 2033 | (67.3) | 687 | (73.9) | <0.001 |
| ≥ Minimum Wage | 986 | (32.7) | 243 | (26.1) |  |
| Sexual Orientation |  |  |  |  |  |
| Homosexual | 1897 | (62.9) | 546 | (58.7) | 0.069 |
| Heterosexual | 268 | (8.9) | 88 | (9.5) |  |
| Bisexual | 853 | (28.3) | 296 | (31.8) |  |
| Gender |  |  |  |  |  |
| Transgender | 414 | (13.7) | 163 | (17.5) | 0.004 |
| Cisgender | 2605 | (86.3) | 767 | (82.5) |  |
| Sexual Role^a^ |  |  |  |  |  |
| Insertive | 1011 | (33.5) | 334 | (36.0) | 0.228 |
| Receptive | 1102 | (36.5) | 341 | (36.7) |  |
| Versatile | 905 | (30.0) | 254 | (27.3) |  |
| Sex work |  |  |  |  |  |
| Yes | 587 | (19.4) | 281 | (30.2) | <0.001 |
| No | 2432 | (80.6) | 649 | (69.8) |  |
| Any Alcohol Use Disorder (AUDIT≥8) | |  |  |  |  |
| Yes | 1905 | (63.1) | 628 | (67.5) | 0.014 |
| No | 1114 | (36.9) | 302 | (32.5) |  |
| Total Number of Male Sex Partners | |  |  |  |  |
| Median (IQR) | 3 | (1-6) | 2 | (1-8) |  |
| No. of stable partners in last 3 months | |  |  |  |  |
| 0 | 1916 | (63.5) | 610 | (65.6) | 0.076 |
| 1 | 731 | (24.2) | 193 | (20.8) |  |
| 2+ | 372 | (12.3) | 127 | (13.7) |  |
